# Chimeric Antigen Receptor (CAR)-NK92 cells effective against glioblastoma, breast- and pancreatic cancer in vitro and in a murine xenograft model of ovarian cancer

**DOI:** 10.1186/s12935-025-03865-0

**Published:** 2025-07-11

**Authors:** Abdelhadi Boulifa, Alexander Sebastian Franzén, Martin J. Raftery, Clarissa Radecke, Gabriele Pecher

**Affiliations:** 1 Berlin Institute of Health at Charité– Universitätsmedizin Berlin, Charitéplatz 1, 10117 Berlin, Germany; 2 Competence Center of Immuno-Oncology and Translational Cell Therapy (KITZ), Department of Hematology, Oncology and Tumor Immunology, CCM, Charité– Universitätsmedizin Berlin, Corporate Member of Freie Universität Berlin and Humboldt-Universität zu Berlin, Charitéplatz 1, 10117 Berlin, Germany

**Keywords:** Glioblastoma, Breast cancer, Pancreatic cancer, Ovarian cancer, CD44v6, CAR-NK, 3D tumor models, NK92 cell line, Immunotherapy

## Abstract

**Background:**

Aggressive tumors such as glioblastoma, breast, ovarian and pancreatic cancer have low survival rates and new therapies are urgently needed. One potential target is CD44v6, a splice variant of CD44 that is associated with poor prognosis. Recently, NK cells expressing CAR molecules have shown promise in combining specific targeting of solid tumors with a low risk of side effects. The aim of the current study is to explore the efficacy of the CD44v6-CAR construct expressed in the NK cell line NK92 against solid tumors both in vitro and in vivo.

**Methods:**

Flow cytometry was used to evaluate the expression of CD44v6 on glioblastoma, breast, ovarian and pancreatic cancer cell lines. In order to investigate the efficacy of CD44v6-CAR-NK92 against these solid tumors in 2D and 3D models, cytotoxicity was measured using a luminescent cell viability assay. Additionally, we assessed the levels of IFN-γ in cell culture supernatants using an ELISA method. Finally, we evaluated our therapeutic in vivo using a xenografted murine model of ovarian cancer through bioluminescent imaging.

**Results:**

CD44v6-CAR-NK92 cells exhibit specific cytotoxicity against glioblastoma, breast, ovarian and pancreatic cancer after 24 h compared to the control, both in 2D and 3D models. Furthermore, the activity of CD44v6-CAR-NK92 was validated by quantifying specific cytokine release in response to target cells. Finally, we could show that CD44v6-CAR-NK92 was effective in reducing tumor burden in a xenografted murine model of ovarian cancer.

**Conclusion:**

Our results demonstrate that CD44v6-CAR-NK92 cells could be an attractive therapeutic agent for the treatment of solid tumors.

**Supplementary Information:**

The online version contains supplementary material available at 10.1186/s12935-025-03865-0.

## Background

Cancer remains a significant global burden with a need for more targeted treatment options. The identification of new cell surface biomarkers plays a crucial role in adapting therapeutic strategies and optimizing patient care for targeted treatment options. Current therapeutic approaches for various cancer types show significant efficacy, especially in the subgroups expressing specific biomarkers or receptors [1, 2]. This is particularly relevant for aggressive tumors such as glioblastoma, Triple Negative Breast Cancer (TNBC), ovarian cancer and pancreatic cancer, which are challenging to treat. Ovarian cancer is the ultimate example of unmet demand: it is the most lethal of gynecologic cancers, diagnosed at > 70% with late-stage disease despite nonspecific presenting symptoms and without early detection strategy [3–5]. Peritoneal seeding and chemoresistance lead to a less than 50% 5-year survival rate [5].The high mortality rate of glioblastomas is roughly 90% within 5 years which underlines the necessity for novel treatment strategies [6]. The International Agency for Research on Cancer (IARC) reports that one of the most common cancer diagnoses worldwide recently is breast cancer, with an estimated 11% new cases reported [7]. Pancreatic cancer has a particularly low 5-year survival rate of around 10% [8]. Given these challenging prognoses, cell therapy has gained importance and shows promise as a targeted treatment option against these aggressive tumors. Cell therapy mobilizes the patient’s immune system to target cancer cells. Particularly promising is Chimeric Antigen Receptor-(CAR)-Natural Killer (NK) cell therapy, which is an innovative strategy to further increase the effectiveness of immunotherapy and target these difficult to treat cancers [9].

The Food and Drug Administration (FDA) approval of CD19-targeted CAR-T cell therapy for hematological cancer was a significant advance in genetically modified cell therapies [10]. However, CAR-T cell therapy has been shown to have lower remission rates in solid tumor patients [11]. Factors contributing to this challenge include immunosuppressive tumor microenvironments, poor T cell infiltration, and antigen heterogeneity [12, 13]. These factors hamper the effectiveness of CAR-T cells by preventing them from effectively recognizing and eliminating cancer cells. As an alternative, NK and CAR-NK cells have demonstrated efficiency in treating hematological malignancies with a low side effect profile and are being explored as a potential option for solid tumor treatment [14–17]. CAR-NK cells have a low risk of Graft-versus-Host Disease (GvHD) and can be engineered to target a variety of tumors, potentially reducing the risk of relapse and improving cancer treatment options [18]. Furthermore, CAR-NK cells are an “off-the-shelf” immunotherapy product which can be produced from different sources and administered to different patients [15–17, 19]. NK cell lines are particularly relevant for CAR-NK therapy as they share the low side effect profile of primary NK cells but overcome some clinical manufacturing challenges, such as achieving clinically relevant cell expansion and gene transfer difficulties. The NK92 cell line, already used in clinical settings, exemplifies this potential. Due to its malignant origin, these cells must undergo irradiation before clinical administration as an added safety measure [20, 21]. NK cell lines are also a crucial tool used to understand NK cell function and biology which can be translated into CAR-NK therapies.

In hematological and epithelial tumors, the hyaluronate receptor CD44, a class I membrane glycoprotein, is overexpressed and is associated with metastases and poor prognosis in many forms of cancers. In Multiple Myeloma (MM) [22] and Acute Myeloid Leukemia (AML) [23], the variant 6 isoform (CD44v6) is upregulated and is associated with poor prognosis. This also applies to solid tumors such as pancreatic, breast, glioblastoma and head and neck cancer, where it contributes to the metastases [24–27]. For instance, CD44v6 has been shown to convert breast cancer cells into a more aggressive phenotype with loss of endocrine response, which may explain its prevalence in hard-to-treat hormone receptor negative TNBC, making CD44v6 an ideal target for CAR-NK cell therapy [28, 29]. Taken together this suggests that CD44v6 can be used as an antigen to target multiple cancer forms including metastases.

We have previously shown that a novel CAR specific for CD44v6 has the potential to efficiently eliminate breast cancer lines [29]. The CAR design contains a CD44v6 recognition domain with the hinge region of IgG1, the transmembrane and signaling domains of CD28 and the signaling domain of CD3ζ. The CAR vector includes the CD44v6 CAR, a checkpoint inhibitor (PD1x), a suicide gene (thymidine kinase of herpes simplex virus type 1; HSV TK) and a response element with an IL-15 superagonist (15R15) controlled by an NFAT promoter [29]. In this study, we aim to investigate the efficacy of the CD44v6-CAR construct in NK92 cells against various solid tumors, including glioblastoma, breast cancer, ovarian cancer, and pancreatic cancer. Our hypothesis is that the NK92 cell line can serve as an effective alternative to primary NK cells, specifically recognizing and killing tumor cells across different cancer types in both 2D and 3D models. Additionally, we aim to validate our hypothesis through in vivo evaluations using a xenografted murine model of ovarian cancer, employing bioluminescent imaging to assess the therapeutic efficacy of the CD44v6-CAR construct. This comprehensive approach offers a broader treatment option for malignancies expressing CD44v6.

## Methods

### Cell culture

Cell culture was performed under sterile conditions. Each cell type was incubated in a CO_2_ incubator at 37 °C, 5% CO_2_ and 95% humidity. The glioblastoma cell lines U-138-MG (DSMZ, Germany) and U-251-MG (Sigma-Aldrich Chemie GmbH), breast cancer cell lines MCF-7, HCC1937, SKBR3 and T47D (DSMZ, Germany), the pancreatic cancer cell line PANC-1 (DSMZ, Germany), and the fibroblast cell line MRC5 (LGC Standards GmbH) were cultured in Roswell Park Memorial Institute (RPMI) 1640 medium (Gibco) supplemented with 10% heat inactivated Fetal Bovine Serum (hiFBS), 1% Penicillin-Streptomycin (PS), while the breast cancer cell line MDA-MB-231 (DSMZ, Germany) and the ovarian cancer cell line SKOV-3 (Merck, Germany) were cultured with Dulbecco’s Modified Eagle’s Medium (DMEM; Gibco) containing 10% hiFBS, 1% PS and 1% Non-Essential Amino Acid (NEAA; Gibco). The glioblastoma cell line GAMG (DSMZ, Germany) was also cultured with DMEM (Gibco) containing 10% hiFBS, 1% PS and 4% NEAA. The cell lines were passaged on a weekly basis and regularly tested for mycoplasma (Venor^®^GeM, Minerva Biolabs).

NK92 cells were cultured in RPMI supplemented with 10% FBS, 1% PS, 1% NEAA, 1% sodium pyruvate and 200 U/mL IL-2 (Immunotools).

### Preparation of 3D cell culture (multicellular tumor spheroids)

In the present study Multicellular Tumor Spheroids (MCTS) were prepared using anti-adherence rinsing solution (Stemcell Technologies), this liquid overlay technique based on previously published protocols [29, 30] forces the cells to adhere to each other forming solid 3D tumor aggregates. Briefly, a 96-well round bottom plate was pre-coated with the anti-adherence rinsing solution, then the glioblastoma, breast cancer and pancreatic cancer cell lines were passaged, counted, and seeded in triplicate at 1 × 10^3^ cells/well for 48 h in an incubator at 37 °C, 5% CO_2_, and 95% humidity.

MRC5 fibroblasts were introduced in a 1:1 ratio with MCF-7 and PANC-1 cells to improve the model of the MCF-7 and PANC-1 cell lines and to obtain uniform MCTS.

### Lentiviral vector generation and cell transduction

Lentiviral vectors were produced by transiently transfecting HEK-293T cells using linear polyethylenimine (PEI). Plasmid DNA was mixed in DMEM at a 5:4:3:1 ratio (CAR: pMDLg: pRSV-Rev: pMD2G) and combined with diluted PEI (1:13, DNA: PEI). After brief vortexing, the mixture was incubated at room temperature for 20 min. Preplated HEK cells were then supplemented with fresh DMEM, and the transfection solution was added dropwise. Following a 3–4-hour preincubation at 37 °C and 5% CO₂, the medium was replaced with complete DMEM containing 10% FBS, and the cells were incubated overnight. Viral supernatants were collected at 12, 24, and 48 h post-transfection, centrifuged at 4 °C and 300× g for 10 min, and filtered through a 0.45-µm syringe filter. The virus was then ultracentrifuged at 25,000× g for 90 min, the pellet resuspended in PBS, aliquoted, flash-frozen, and stored at − 80 °C, with a portion reserved for viral titration. To perform transduction, target cells were resuspended in virus-containing medium at a MOI of 10 or more with 5 µg/mL polybrene, incubated for 60 min on a rotating mixer at room temperature with a subsequent spin inoculation step at 800× g for 120 min. They were then incubated in a standard cell culture environment overnight and medium changed the next morning.

### Cytotoxicity assays

The CellTiter-Glo^®^ 2.0 assay (Promega) was performed according to the manufacturer’s protocol and used to measure cytotoxicity of CAR-NK92 cells against various solid tumors. The assay principle is based on the measurement of the amount of ATP present, which determines the number of living cells. The effector and target cells were incubated together at the indicated E: T ratios for 18 h and 24 h. After incubation, supernatant was removed for subsequent Enzyme-Linked Immunosorbent Assay (ELISA) measurement and the CellTiter-Glo^®^ reagent was added according to the manufacturer’s protocol and then measured using a Tristar 3 multimode plate reader (Berthold Technologies). The cytotoxicity of the NK cells was calculated based on the ATP released by the target and effector cells according to the following formula:

$$\eqalign{& Cytotoxicity{\rm{ }}\left( \%  \right){\rm{ }} =   \cr & \,\,\left( {100 - {{Sample{\rm{}}release - Effector{\rm{}}release{\rm{}}} \over {Targetrelease{\rm{}}}}} \right) \times 100 \cr} $$

### Enzyme-linked immunosorbent assay (ELISA)

Proteins contained in a solution can be specifically detected and quantified using an ELISA. In this study, this method was used to determine Interferon-gamma level (IFN-γ) in cell culture supernatants. The assay was performed with ELISA MAX™ Deluxe Set Human IFN-γ (BioLegend) according to the manufacturer’s protocol and the Optical Density (OD) was measured at a wavelength of 450 nm. A standard curve of OD is generated using known concentrations. The OD of an unknown sample can then be compared to the standard curve to determine its concentration. The data is presented as concentrations (pg/ml) according to the formula determined from the standard curve.

### Flow cytometry

For flow cytometry, the cells were pelleted and washed once with Phosphate Buffered Saline (PBS; Gibco). The cells were then resuspended, stained with fluorescent antibodies and incubated at 4 °C for 30 min. After incubation, the cells were washed at least once with PBS, resuspended in PBS with 0.3% formaldehyde and measured in a flow cytometer (FACScalibur BD). Anti-CD44v6 PE (clone 2F10) was supplied by R&D Systems, Minneapolis, MN, USA. IgG Fc (clone QA19A42), Streptavidin PE and AF647 were supplied by Biolegend (San Diego, CA, USA). Polyclonal anti-IgG Fc was supplied by Jackson Immunoresearch, West Grove, PA, USA. Fluorescence was measured using a BD FACScalibur™ and data were analysed using FlowJo v10.8 (Becton Dickinson and Company, Franklin Lakes, NJ, USA; 2019).

### Murine xenograft model of human ovarian carcinoma

Six- to eight-week-old female NOG mice were obtained from Taconic (Denmark). All animal experiments were conducted by EPO Berlin-Buch GmbH (Germany), a facility fully accredited by American Association for Accreditation of Laboratory Animal Care (AAALAC). The studies were performed in accordance with the United Kingdom Coordinating Committee on Cancer Research (UKCCCR) guidelines for animal welfare, the German Animal Protection Law and current ethical standards of local authorities. Experimental protocols were approved by the local authority (LaGeSo, Berlin, E0023/23).

The antitumor efficacy of NK92 CAR cells was assessed using a human ovarian carcinoma cell-derived xenograft model. Modified CD44v6-SKOV-3/luc cells (1 × 10⁶) suspended in 100 µl PBS were intraperitoneally (i.p.) inoculated into 42 female NOG mice on day 0. Mice were divided into 3 groups, PBS treatment, mock treatment (Empty CAR NK92 cells with a non-binding CAR) and CAR treatment (CD44v6-CAR transduced NK92 cells). On days 4 and 11, the experimental groups received either PBS or irradiated NK-92 CAR cells (10 Gy). NK-92 cells were irradiated using a gamma irradiator set to deliver 10 Gy over 40 min, a dose previously determined to be sufficient for prevention of proliferation but retention of functionality after storage by freezing. Following irradiation, NK-92 cells were cryopreserved until the time of administration. Before injection, the cells were thawed, resuspended in NK-MACS medium (without supplements), and transported on ice to the animal facility. The final dose of NK-92 cells was 1 × 10⁷ cells in 100 µL, administered via intraperitoneal (i.p.) injection. To monitor overall health and potential treatment-related toxicity, body weights were recorded three times per week. Tumor progression was assessed using Bioluminescence Imaging (BLI).

### Statistical analysis

Statistical analysis was performed using Prism (v. 8.4.2, Graphpad). The data were compared using the Two-way ANOVA and multiple t test. The error bars show the standard error of the mean ± SEM. Statistical significances are marked *p* < 0.05 = *, *p* < 0.01 = **, *p* < 0.001 = ***, *p* < 0.0001 = **** and non-significance is marked with ns (*p* ≥ 0,05).

## Results

**Fig. 1:**
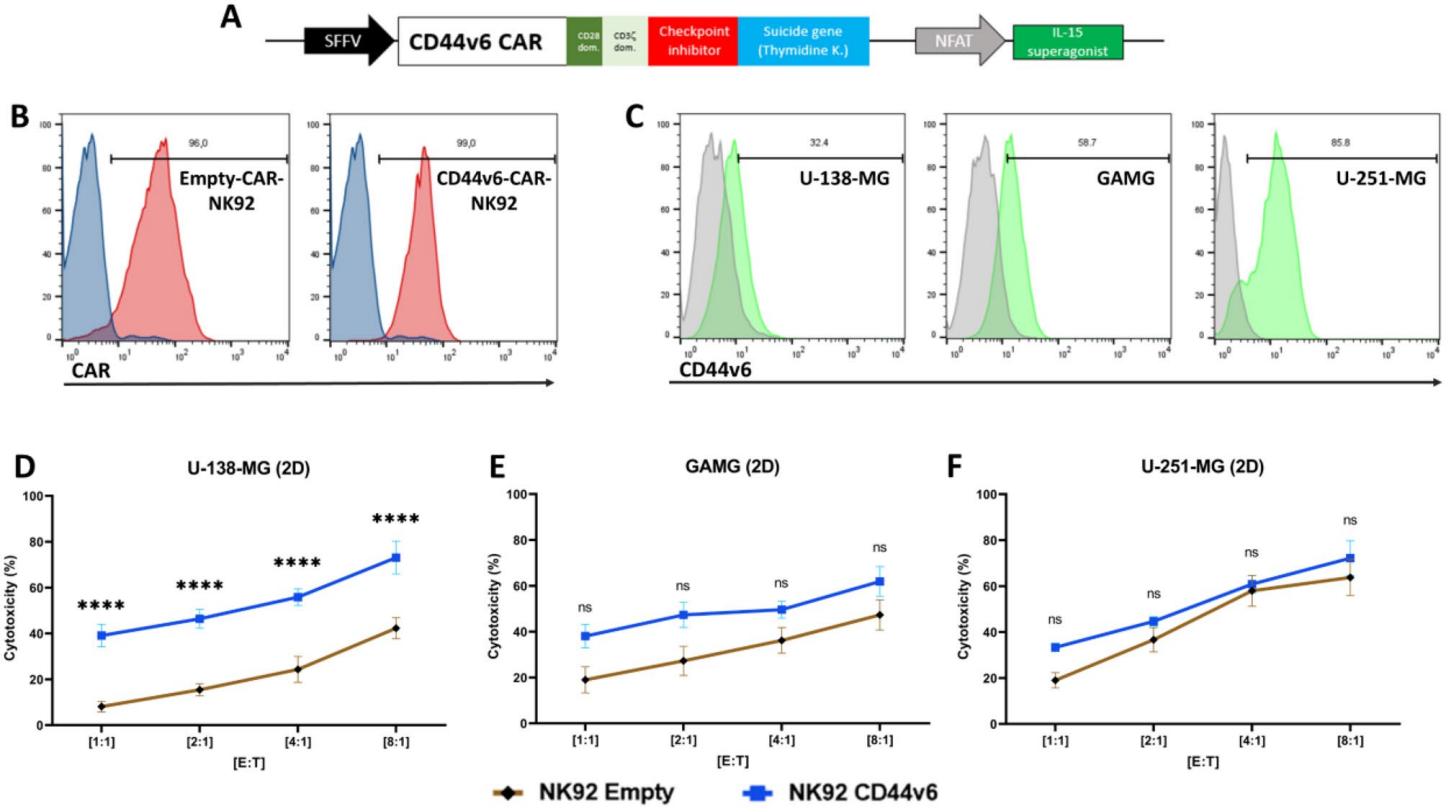
2D model of cytotoxicity efficacy of CD44v6-CAR-NK92 against glioblastoma: (**A**) Schematic representation of the CD44v6-specific CAR construct. (**B**) Representative flow cytometric analysis of CAR expression in transduced NK92 cells. The control staining is represented by the blue curves and the red curves represent the staining of the target molecule. (**C**) Representative flow cytometric analysis of CD44v6 expression on the glioblastoma tumor cell lines GAMG, U-138-MG and U-251-MG. (**D-F**) Cytotoxicity assays showing the percent cytotoxicity of CD44v6-CAR-NK92 cells against glioblastoma cell lines in the 2D model compared to Empty-CAR-NK92 cells at various effector-to-target ratios (E: T ratios) after 24 h of incubation. The results represent *n* = 3 independent experiments performed in triplicate. Statistical significances are presented using two-way ANOVA and marked *p* < 0.05 = *, *p* < 0.01 = **, *p* < 0.001 = ***, *p* < 0.00001 = ****, and ns for non-significant p-values

To investigate the efficacy of CD44v6-CAR construct in NK92 cells against various solid tumors such as glioblastoma, breast cancer, ovarian cancer and pancreatic cancer, we transduced NK92 cells with CD44v6 CAR (CD44v6-CAR-NK92) or a control construct lacking the CD44v6 recognition domain (Empty-CAR-NK92) (Fig. 1A). The expression of the CD44v6 CAR and the control construct lacking the CD44v6 recognition domain were confirmed by flow cytometry using a fluorescently labeled anti-IgG Fc (Fig. 1B).

### CD44v6 CAR effective against glioblastoma cell lines

In order to test the efficacy of CD44v6-CAR-NK92 cells against glioblastoma, we used three glioblastoma cell lines (GAMG, U-138-MG, U-251-MG). Initially, we confirmed the expression of CD44v6 on the cell surface (Fig. 1C). Followed by cytotoxicity testing of CD44v6-CAR-NK92 cells against the identified glioblastoma cell lines in a 2D cell culture model. The results showed that CD44v6-CAR-NK92 cells have significant cytotoxicity against U-138-MG (2D) after 24 h incubation compared to the control (Empty-CAR-NK92) in all tested E: T ratios (Fig. 1D), whereas in 2D culture the cytotoxicity of CD44v6-CAR-NK92 cells against GAMG (Fig. 1E) and U-251-MG (Fig. 1F) cells showed increased but non-significant cytotoxic activity compared to Empty-CAR-NK92 cells. The small difference in cell killing in GAMG and U‑251‑MG cell lines in 2D cultures has been observed consistently in numerous independent experiments. This is a result of the inherent NK92 cell killing under 2D culture conditions, which masks the additional CAR cell killing effect that becomes more apparent in 3D models [31].

We then tested CD44v6-CAR-NK92 cells against glioblastoma cell lines in a MCTS model (3D). Compared to 2D models, 3D MCTS models offer a more functional and realistic platform for studying drug effects and tumor biology. They allow for better reproduction of the tumor microenvironment, more convenient drug testing and more detailed study of the immune response [32].

**Fig. 2:**
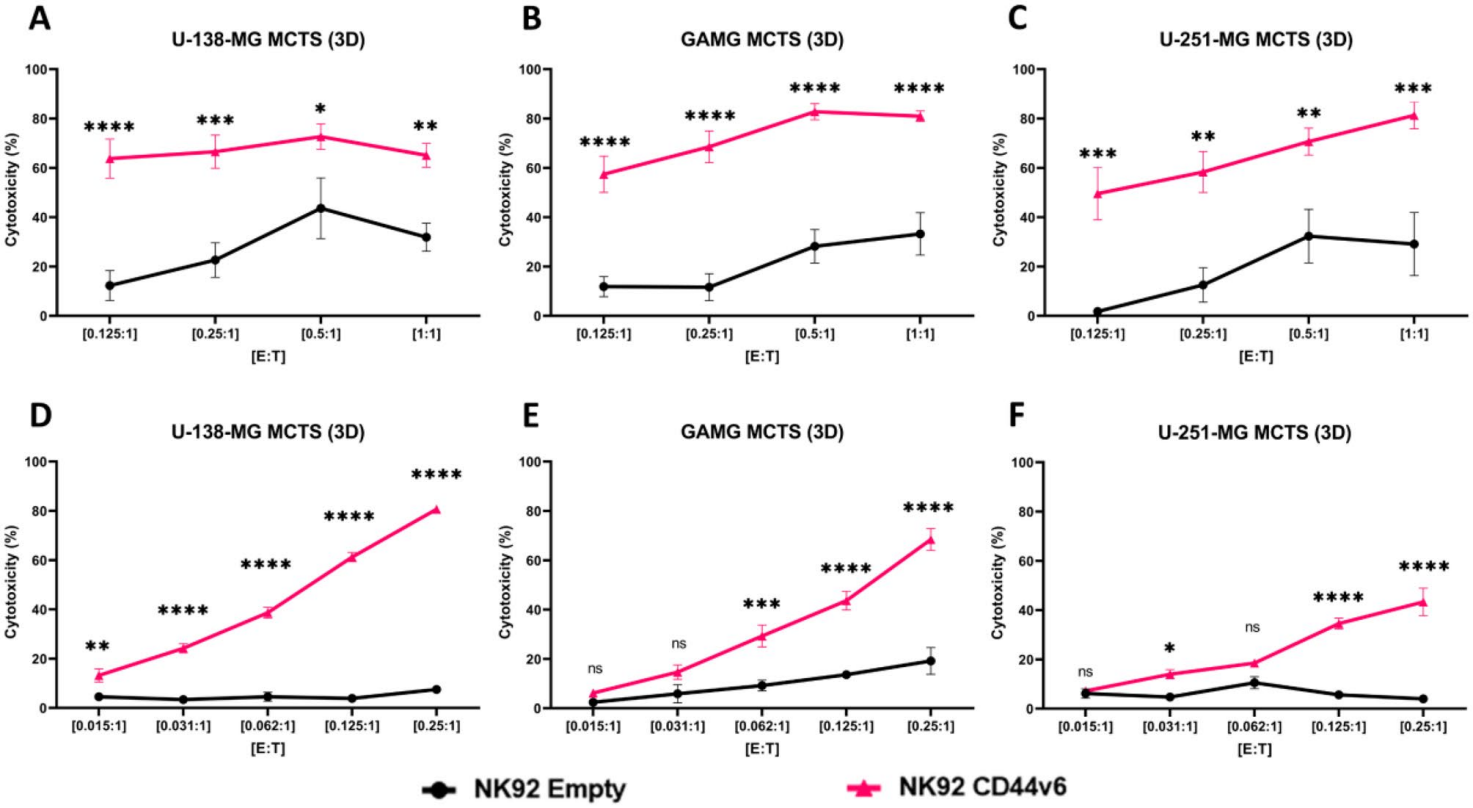
3D model of cytotoxicity efficacy of CD44v6-CAR-NK92 against glioblastoma: (**A-C**) Cytotoxicity assays showing the percent cytotoxicity of CD44v6-CAR-NK92 cells against glioblastoma cell lines in the 3D model compared to Empty-CAR-NK92 cells at various effector-to-target ratios (E: T ratios) from [1:1] to [1:0.125] after 24 h of incubation. (**D-F**) Cytotoxicity assays showing the percent cytotoxicity of CD44v6-CAR-NK92 cells against glioblastoma cell lines in the 3D model compared to Empty-CAR-NK92 cells at various effector-to-target ratios (E: T ratios) from [1:0.25] to [1:0.015] after 24 h of incubation. The results represent *n* = 3 independent experiments performed in triplicate. Statistical significances are presented using two-way ANOVA and marked *p* < 0.05 = *, *p* < 0.01 = **, *p* < 0.001 = ***, *p* < 0.00001 = ****, and ns for non-significant p-values

We could show the cytotoxic effect of CD44v6-CAR-NK92 cells against glioblastoma cell lines in the 3D model compared to Empty-CAR-NK92 cells (Fig. 2A-F). In contrast to the 2D model the cytotoxicity of CD44v6-CAR-NK92 cells was highly significant against all lines tested in the 3D model, U-138-MG MCTS (Fig. 2A), GAMG MCTS (Fig. 2B) and U-251-MG MCTS (Fig. 2C) at all E: T ratios compared to Empty-CAR-NK92 cells, where the cytotoxicity reached at the highest ratio [1:1] of approximately 80% for all cell lines. We observed that the cytotoxicity of CD44v6-CAR-NK92 cells at the lowest ratio [0.125: 1] against U-138-MG MCTS, GAMG-MCTS and U-251-MG MCTS increased by approximately 64%, 58% and 50%, respectively. As all cell lines tested in 3D culture showed a highly significant cytotoxicity at all E: T ratios we performed additional experiments with even lower E: T ratios to further evaluate the efficiency of CD44v6-CAR-NK92 cell activity and to test whether CD44v6-CAR-NK92 cells are able to eliminate glioblastoma MCTS even under these suboptimal conditions.

We observed that CD44v6-CAR-NK92 cells demonstrated their ability to target and kill glioblastoma cell lines in 3D models even at high dilution. They showed significant killing abilities against U-138-MG MCTS (Fig. 2D) at all E: T ratios, against GAMG MCTS (Fig. 2E) at the lowest ratio of [0.062:1], and against U-251-MG MCTS (Fig. 2F) at the lowest ratio of [0.031:1] after 24 h compared to the control. In total, we could show highly effective killing of glioblastoma lines with the CD44v6-CAR-NK92.

In line with these observations, while the 2D model showed variable cytotoxicity across glioblastoma cell lines, the 3D model consistently demonstrated potent and highly significant CAR-specific cytotoxicity. This discrepancy may be attributed to the inherent non-specific cytotoxicity of NK92 cells in 2D cultures, which is minimized in 3D models due to the more physiologically relevant tumor microenvironment.

The enhanced cytotoxicity of CD44v6-CAR-NK92 cells in 3D spheroids reflects the biological relevance of such systems in accurately modeling important TME interactions. Unlike 2D monolayers, 3D spheroids reproduce the spatial organization of tumors, where cell-cell and cell-matrix interactions determine immune cell infiltration and effector function. This is particularly relevant in desmoplastic tumors, where cells form dense stromal barriers inhibiting immune cell entry [33]. In addition, 3D models simulate hypoxia and nutrient gradients that induce immunosuppressive factors such as TGF-β, which simulate clinical tumor metabolic stress [34, 35]. The efficient CAR-dependent cytotoxicity in 3D models even at low effector-to-target ratios suggests that CD44v6-CAR-NK92 cells can overcome TME mechanisms of resistance.

### CD44v6-CAR effective against breast Cancer cell lines

**Fig. 3:**
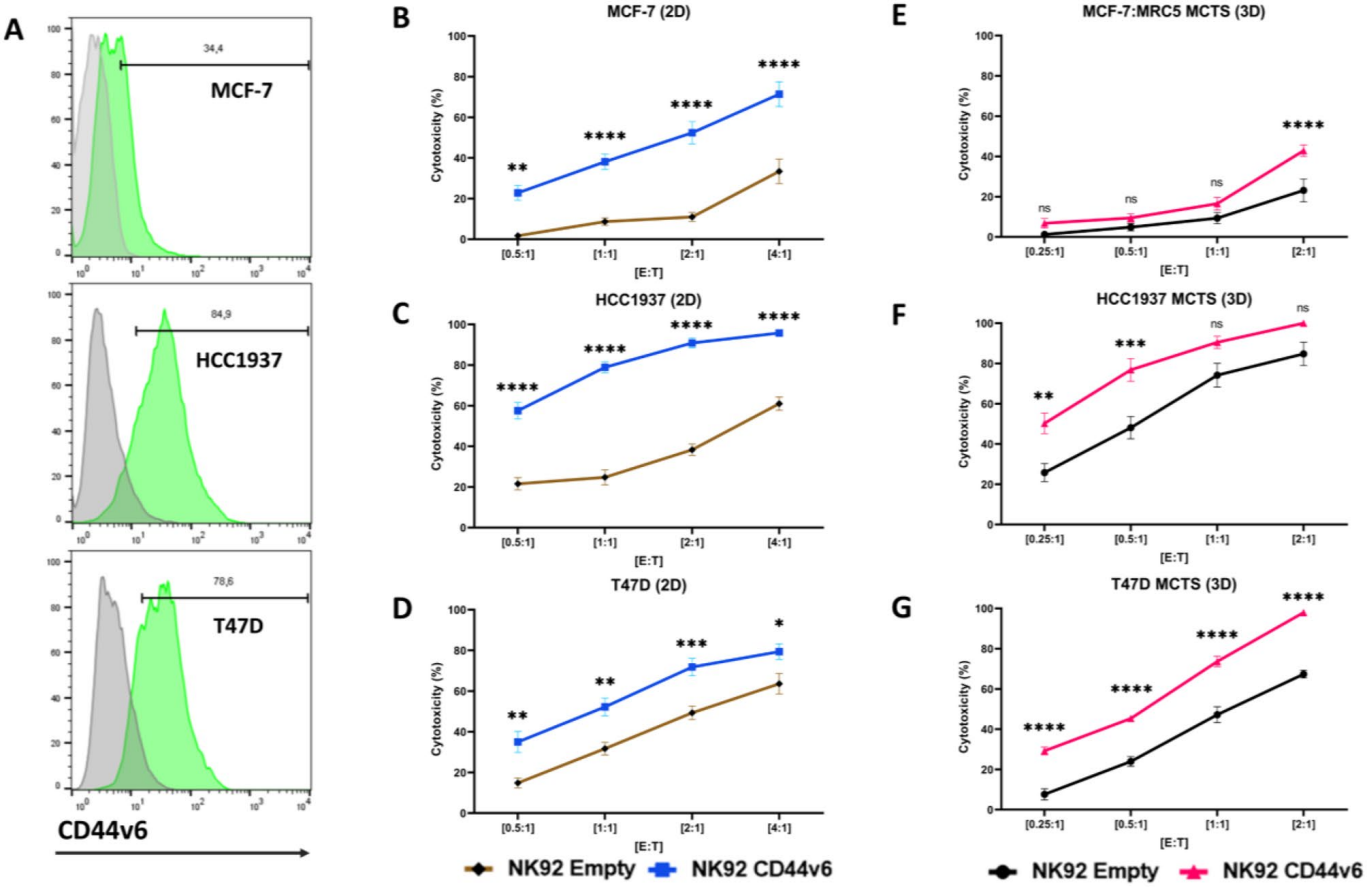
2D and 3D model of cytotoxicity efficacy of CD44v6-CAR-NK92 against breast cancer: (**A**) Representative flow cytometric analysis of CD44v6 expression on the breast cancer cell lines MCF-7, HCC1937 and T47D. (**B-D**) Cytotoxicity assays showing the percent cytotoxicity of CD44v6-CAR-NK92 cells against breast cancer cell lines in the 2D model compared to Empty-CAR-NK92 cells at various effector-to-target ratios (E: T ratios) after 18 h of incubation (**E-G**) Cytotoxicity assays showing the percent cytotoxicity of CD44v6-CAR-NK92 cells against breast cancer cell lines in the 3D model compared to Empty-CAR-NK92 cells at various effector-to-target ratios (E: T ratios) after 24 h of incubation. The results represent *n* = 3–4 independent experiments performed in triplicate. Statistical significances are presented using two-way ANOVA and marked *p* < 0.05 = *, *p* < 0.01 = **, *p* < 0.001 = ***, *p* < 0.00001 = ****, and ns for non-significant p-values

Another malignancy that has been shown to express CD44v6 are breast cancers. Therefore, we investigated the cytotoxicity of the CD44v6-CAR-NK92 cell line against different breast cancer cell lines in 2D and 3D culture models. Using flow cytometry, we confirmed that the breast cancer cell lines MCF-7, HCC1937, T47D, SKBR3, and MDA-MB-231 expresses CD44v6 (Fig. 3A, Supplementary Fig. 1A) making them good targets to study the CD44v6-CAR-NK92 cells efficiency.

The results show that CD44v6-CAR-NK92 cells are able to exert significant cytotoxic effects on all five breast cancer cell lines in 2D model after 18 h at all E: T ratios compared to Empty-CAR-NK92 cells (Fig. 3B, C, D, Supplementary Fig. 1B, C), highlighting the potential of this immunotherapeutic method. We then used 3D models for breast cancer to better simulate the microenvironment and provide a more realistic assessment of the cytotoxic effect of CD44v6-CAR-NK92 cells against MCF-7: MRC5 MCTS, HCC1937 MCTS, and T47D MCTS. The results from the 3D cultures confirmed the effective cytotoxicity of CD44v6-CAR-NK92 cells against MCF-7: MRC5 MCTS (Fig. 3E), HCC1937 MCTS (Fig. 3F), and T47D MCTS (Fig. 3G) compared to Empty-CAR-NK92 cells, indicating that the interaction between the CD44v6-CAR-NK92 cells and the tumor microenvironment plays a role in the effectiveness of the therapy.

### CD44v6-CAR effective against pancreatic Cancer cell lines

**Fig. 4:**
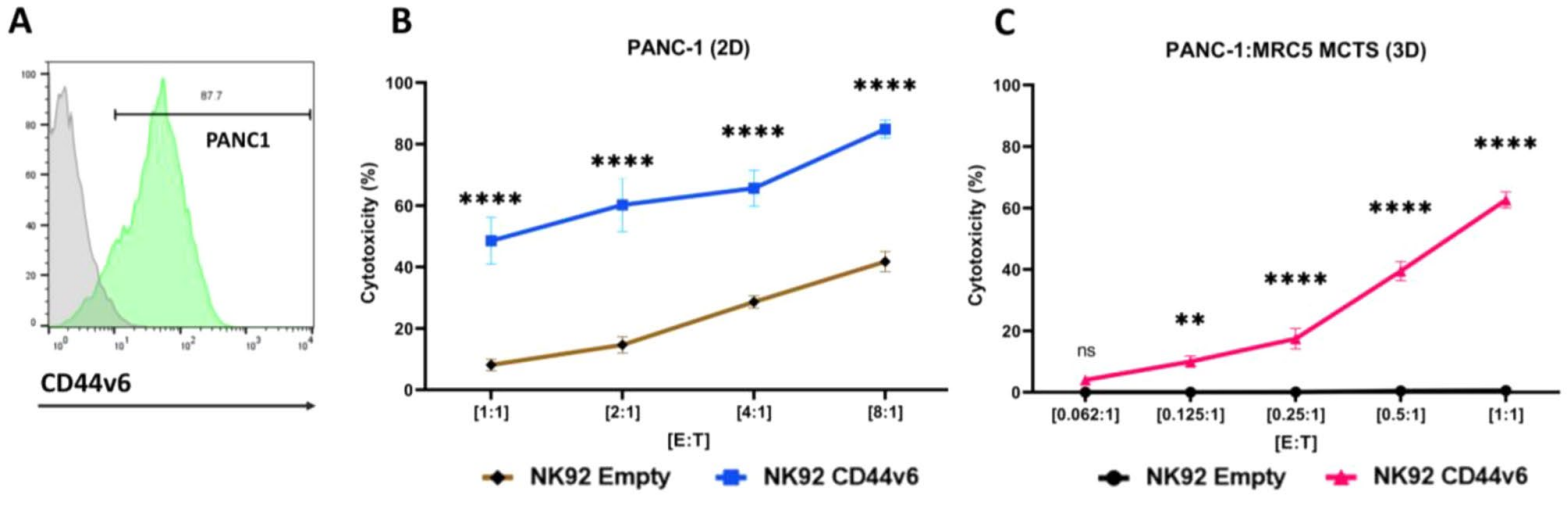
2D and 3D-model of cytotoxicity efficacy of CD44v6-CAR-NK92 against pancreatic cancer: (**A**) Representative flow cytometric analysis of CD44v6 expression in the pancreatic cancer cell line PANC-1. (**B-C**) Cytotoxicity assays showing the percent cytotoxicity of CD44v6-CAR-NK92 cells against pancreatic cancer cell line in the 2D- and 3D-model compared to Empty-CAR-NK92 cells at various effector-to-target ratios (E: T ratios) after 24 h of incubation. The results represent *n* = 3 independent experiments performed in triplicate. Statistical significances are presented using two-way ANOVA and marked *p* < 0.05 = *, *p* < 0.01 = **, *p* < 0.001 = ***, *p* < 0.00001 = ****, and ns for non-significant p-values

We identified pancreatic cancer as a third malignancy with elevated CD44v6 levels and confirmed that the PANC-1 cell line exhibits increased CD44v6 expression (Fig. 4A). This led us to investigate the cytotoxic effects of CD44v6-CAR-NK92 cells on PANC-1 in both 2D and 3D models. To support the formation of 3D solid structures, PANC-1 cells were cultured with fibroblasts, which facilitated the creation of uniform 3D models for cytotoxicity assays. To ensure specificity of the cytotoxic effect in 3D models, we performed flow cytometry staining of CD44v6 on MRC5 fibroblasts, which confirmed that these fibroblasts expressed almost no CD44v6 (Supplementary Fig. 1A).

Our results showed that CD44v6-CAR-NK92 cells exhibited significant cytotoxicity against the PANC-1 cell line in 2D models after 24 h at all E: T ratios compared to Empty-CAR-NK92 cells (Fig. 4B). Furthermore, we also showed that CD44v6-CAR-NK92 cells exhibit significant killing capacity against co-culture PANC-1:MRC5 MCTS after 24 h compared to Empty-CAR-NK92 cells (Fig. 4C).

### Immune response triggering of CD44v6-CAR-NK92 cells

**Fig. 5:**
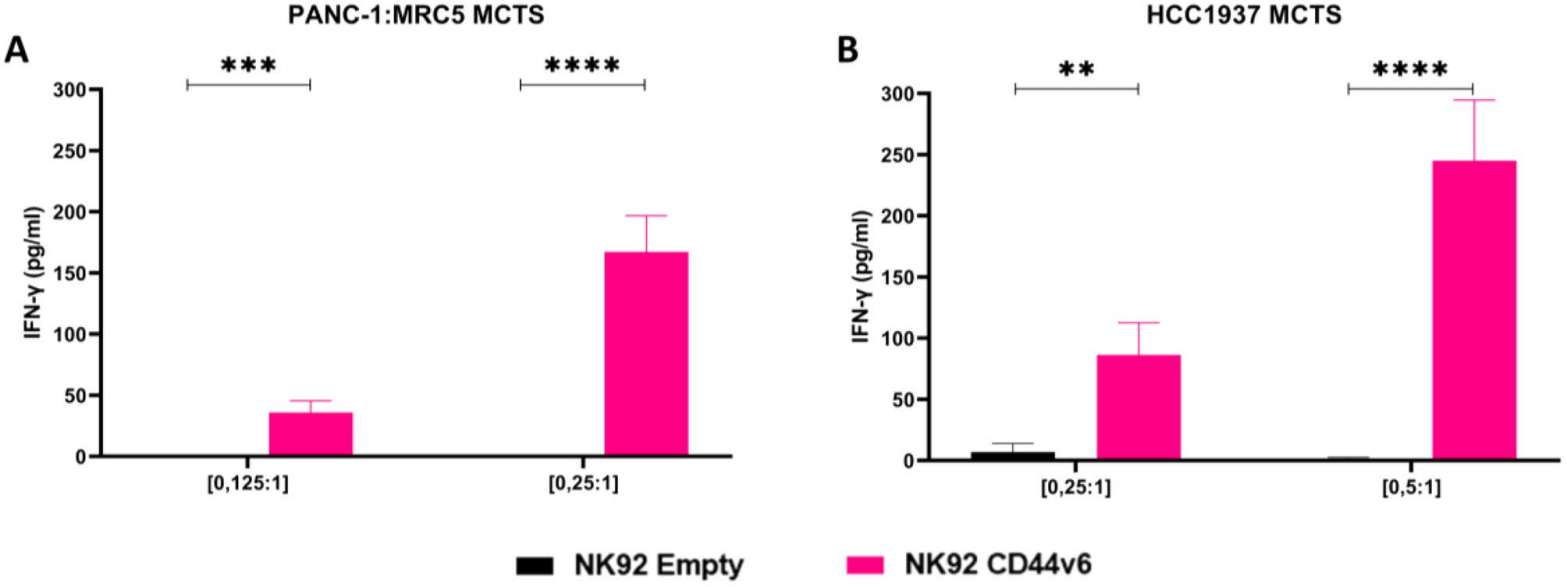
Enzyme-linked immunosorbent assay (ELISA): ELISA used to analyze the release of IFN-γ by CD44v6-CAR-NK92 cells or Empty-CAR-NK92 cells in co-culture supernatants against (**A**) PANC-1:MRC5 MCTS and (**B**) HCC1937 MCTS. The results represent *n* = 3 independent experiments performed in triplicate. Statistical significances are presented using multiple t test and marked *p* < 0.05 = *, *p* < 0.01 = **, *p* < 0.001 = ***, *p* < 0.00001 = ****, and ns for non-significant p-values

We used an ELISA to quantify the immune response of CD44v6-CAR-NK92 cells against breast cancer and pancreatic cancer cell lines. This examined the concentrations of IFN-γ released by the NK92 cell line. The characterization of the activity and efficacy of NK cells depends largely on this cytokine. It plays an important role in the immune response and indicate the cytotoxic activity of CD44v6-CAR-NK92 cells. To confirm the activity of CD44v6-CAR NK92 cells, we investigated the supernatant of the lowest E: T ratio that exhibited significant cytotoxicity in the 3D models of breast and pancreatic cancer. The results indicate a significant increase in IFN-γ production in PANC-1-MRC5 MCTS (Fig. 5A) and HCC1937 MCTS (Fig. 5B) treated with CD44v6-CAR-NK92 cells. The production of IFN-γ in CD44v6-CAR-NK92 cells was significantly higher at all E: T ratio in PANC-1-MRC5 MCTS compared to Empty-CAR-NK92 cells. A similar pattern was seen in HCC1937 MCTS, where IFN-γ production was significantly higher in CD44v6-CAR-NK92 cells at all E: T ratio compared to Empty-CAR-NK92 cells. These results show that CD44v6-CAR-NK92 cells support the immune response through IFN-γ secretion and are a promising therapeutic agent for the treatment of solid tumors.

### CD44v6-CAR-NK92 effective in xenografted murine model of ovarian cancer

**Fig. 6:**
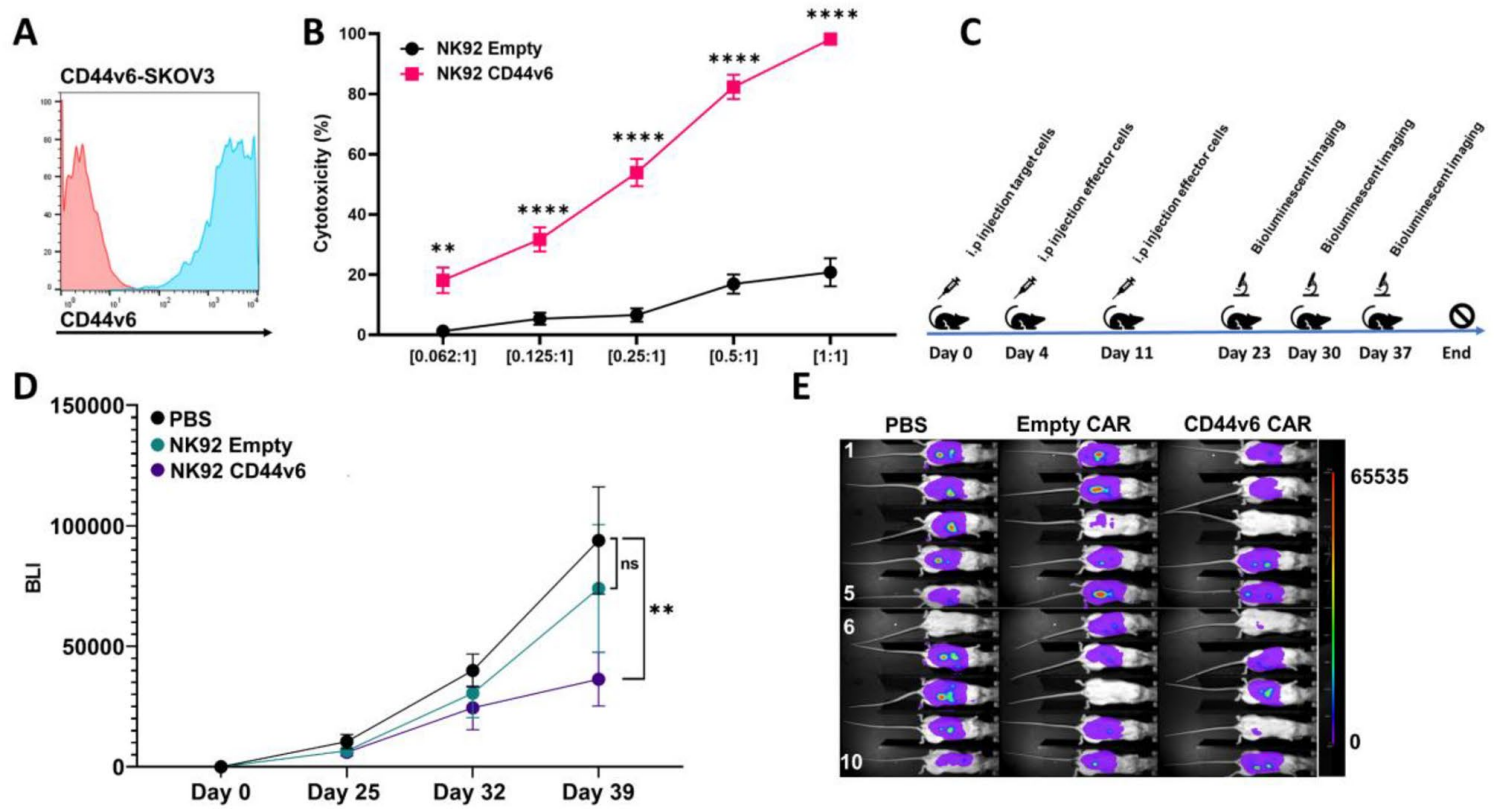
CD44v6-CAR-NK92 xenografted murine model of ovarian cancer SKOV-3 and 3D-model: (**A**) Representative flow cytometry analysis of CD44v6-SKOV-3 cells, illustrating the high expression levels of CD44v6 in this cell line. (**B**) Cytotoxicity assays showing the percentage cytotoxicity of CD44v6-CAR-NK92 cells against CD44v6-SKOV-3 3D tumor models compared to Empty-CAR-NK92 cells across various effector-to-target ratios after 24 h of incubation. Data represent the mean ± SEM from three independent experiments performed in triplicate. (**C**) Graphical overview of the in vivo study design, including a timeline with key intervention points. (**D**) Bioluminescence imaging (BLI) data illustrating tumor growth dynamics for each treatment group. Each group included 10 xenografted mice (*n* = 10). (**E**) Tumor burden analysis for each treatment group at day 39, as assessed by BLI. Statistical significances are presented using two-way ANOVA and marked *p* < 0.05 = *, *p* < 0.01 = **, *p* < 0.001 = ***, *p* < 0.00001 = ****, and ns for non-significant p-values

After confirming the efficacy of CD44v6-CAR-NK92 cells in eliminating 3D solid tumor models expressing CD44v6 across various cancer types, we proceeded to evaluate their performance in an in vivo xenograft mouse model. To this end, we used CD44v6-expressing SKOV-3 ovarian carcinoma cell line (Fig. 6A). CD44v6-CAR-NK92 cells demonstrated robust elimination of 3D solid tumor models derived from CD44v6-SKOV-3 cells across all tested E: T ratios (Fig. 6B). For in vivo experiments, 1 × 10⁶ CD44v6-SKOV-3 cells were injected intraperitoneally (i.p.) into NOG mice. The xenografts received two doses of 1 × 10⁷ irradiated CD44v6-CAR-NK92 cells on days 4 and 11 post-tumor cell injection (Fig. 6C). This treatment schedule was designed to mimic the protocol used in clinical trials for NK92-based therapies, where NK92 cells are irradiated with 10 Gy before cryopreservation, and thawed just prior to administration. Injections of NK92 cells were well tolerated, with no significant body weight loss observed (Supplementary Fig. 2). Bioluminescence analysis revealed a reduction in tumor growth in the CD44v6-CAR-NK92 treated group, with a statistically significant difference compared to the PBS-treated control group on day 39 (Fig. 6D). However, no significance was observed when comparing the CD44v6-CAR-NK92 to the NK92 Empty CAR group. In addition, 3 out of 10 xenografts had almost no tumor burden left at day 39 in the CD44v6 CAR treatment group compared to the Empty CAR group and the PBS group (Fig. 6E). At the study end point the dissection of the mice proved that all mice had developed tumors, however the measurable therapeutic effect of the treatment group could only be observed up till day 39. These results suggest that CD44v6-CAR-NK92 cells fulfill their anti-tumor function against solid tumors in vivo, however an optimized treatment schedule is needed in order to improve the therapeutic efficiency.

The selection of an ovarian cancer model is particularly appropriate because of the disease’s aggressive nature and unfavorable prognosis. Ovarian cancer remains one of the leading causes of cancer mortality in women, owing to the limited early detection methods and effective treatments available [36]. Therefore, our findings underscore the urgent need for new treatment options in ovarian cancer and prompt the continued investigation of the clinical use of CD44v6-CAR-NK92 cell therapy.

## Discussion

Cancer is an urgent problem that is increasing worldwide, causing one in six deaths and leading to a high rate of both morbidity and mortality [37]. Glioblastoma is considered one of the most common malignant primary tumors of the central nervous system with a poor prognosis for patients [38]. Breast and ovarian cancer is responsible for over 2 million new cases each year, making it the leading cause of cancer mortality in women worldwide (6.6%) [39]. Moreover, pancreatic cancer is one of the deadliest solid malignancies due to its aggressiveness. This makes it a persistent global health burden and has a five-year survival rate lower than 5% [40].

Despite the fact that most CAR-T cell clinical trials have shown the treatment’s effectiveness, major adverse effects have been documented [41]. NK cells represent a promising alternative to T cells with fewer reported adverse effects. Several recent studies have demonstrated the efficacy of CAR-NK cells in a therapeutic setting for the elimination of malignant cells [42].

CD44v6 is a promising target molecule that is expressed on the surface of many tumor cell types, including glioblastoma, breast, ovarian and pancreatic cancer [43–45]. By activating signaling pathways that drive tumor growth, migration, and invasion, CD44v6 promotes aggressive tumor behavior [46]. With its increased expression being linked to tumor metastasis and poor prognosis [24, 25]. Expression of CD44v6 on normal primary cell lines such as fibroblasts is nugatory (see supplementary Fig. 1A), making it an attractive cancer target. In this study, we tested a CD44v6-specific CAR construct [29] for efficacy in an NK cell line, NK92 which offers advantages for manufacture of therapeutics and regulatory standardization in cancer therapy. CD44v6-CAR-NK92 cells were tested against various solid tumors, including glioblastoma, breast, ovarian and pancreatic cancer in 2D and 3D models.

We could demonstrate that CD44v6-CAR-NK92 cell exhibited significant cytotoxicity against glioblastoma cell lines in 2D and 3D models compared to the control. This is in line with recent reports [47]. In addition, we observed that our CD44v6-CAR-NK92 cells demonstrated enhanced cytotoxicity in 3D models even at low E: T ratios. Interestingly, this suggests that our CD44v6 CAR-NK92 cells effectively target tumor cells even under unfavorable conditions. This effect may be due to the incorporated IL-15 element in the CD44v6-CAR, which is activated upon binding to its antigen [29]. The resulting IL-15 production minimizes exhaustion and enables NK cells to replenish their granules, facilitating multiple serial killing events against cancer cells [48, 49]. The demonstrated cytotoxicity of CD44v6-CAR-NK92 cells against glioblastoma cell lines in 3D models suggests strong potential for clinical applications, building on the proven effectiveness of CAR-NK cells in complex tumor microenvironments observed in preclinical and early clinical studies [50, 51].

Similar results were obtained by using CD44v6-CAR in primary NK cells directed against TNBC [29]. Furthermore, CD44v6 CAR-NK cells were shown to exhibit a two- to three-fold increase in killing efficiency against breast cancer cell lines in 2D and 3D models. The improved cytotoxicity was observed in breast cancer cell lines with high and low CD44v6 expression, demonstrating the broad applicability of this approach.

Moreover, it has been reported that elevated CD44v6 levels in pancreatic cancer patients have higher metastasis and shorter survival times [52]. Prompting us to investigate the cytotoxic effect of CD44v6-CAR-NK92 cells on the pancreatic cancer cell line PANC-1 in 2D and 3D models. Our results demonstrated significant effectiveness in all the models used and highlighted CD44v6 as a promising target for immunotherapy in pancreatic cancer. These results confirm the previous studies that CAR-NK cells offer a promising way to fight pancreatic cancer by overcoming the immunological obstacles at the tumor microenvironment and enhancing the immune response [53, 54]. Furthermore, CAR-NK cells have been shown to reduce tumor development and increase survival rates in pancreatic cancer mouse models without producing systemic toxicity highlighting the possibility of CAR-NK cell therapy as a successful pancreatic cancer treatment modality [55].

The interaction between CD44v6-CAR-NK92 cells and the surface receptors on tumor cells generates the production of IFN-γ, which can induce apoptosis on the tumor cells making IFN-γ an important cytokine in the immune response against cancer. IFN-γ is produced by NK and T cells, and helps to activate macrophages, increase antigen presentation and induce the cytotoxic effect of immune cells [56]. In this study, we demonstrated that CD44v6-CAR-NK92 cells have increased IFN-γ production when interacting with PANC-1-MRC5 MCTS and HCC1937 MCTS, suggesting that CD44v6-CAR-NK92 cells can induce a strong immune response against the tumor cells [57]. Taken together these results in combination with the demonstrated cytotoxicity against 3D solid tumor cancer models underline the efficacy of CAR-NK cells and demonstrate the ability to muster a robust immune response even at low dosages. Furthermore, while a broader cytokine analysis could provide additional insights, the data we observed strongly support the notion of CAR-mediated cytotoxicity rather than non-specific activation.

Our CD44v6-CAR construct already incorporates a thymidine kinase (HSV-TK) suicide gene, providing a built-in safety mechanism that enables pharmacologically controlled elimination of the CAR-NK92 cells if needed [29]. This suicide gene system has been widely validated in clinical applications, allowing selective depletion of genetically modified cells upon administration of ganciclovir. Although this design considerably enhances safety with an immediate fail-safe option in the case of unexpected toxicities, the HSV-TK system creates a way for controlled ablation of the cells. Future modifications could incorporate small-molecule-regulated ON/OFF switches for added safety features, as such systems previously have been tried in other CAR-based immunotherapies with success [58].

The in vivo efficacy of CD44v6-CAR-NK92 cells demonstrated a reduction of tumor burden, with the primary effect observed 28 days after the second injection of effector cells. However, discrepancies were observed in the in vivo data that were not present in the in vitro data that requires further discussion. The observed reduction in the tumor burden could only be seen between the PBS control group and not when comparing the effect between the Empty CAR group and the CD44v6 CAR group. This is likely explained by the irradiation process that is known to affect the cytotoxic response of NK92 cells. Irradiation of NK92 is a safety measure in clinically used cells and therefore exhibit a short in vivo lifespan, with their antitumor effects primarily manifesting shortly after infusion [20, 59]. In order to better test the in vivo efficacy of the irradiated NK92 cells a more intense administration scheme may be required to see an increased effect of the CD44v6 CAR in reducing the tumor burden in vivo.

Our study, alongside others, has demonstrated that NK92 cell injections are well-tolerated in xenograft models, suggesting that an intensified injection schedule is feasible. Additionally, studies have shown that cytokine support can prolong the longevity and enhance the efficacy of NK92 cells in vivo, making this a viable strategy for improving antitumor responses in future study designs [60, 61]. Our findings confirm that CD44v6-CAR-NK92 cells exhibit in vivo functionality against solid tumors, underscoring their potential as a therapeutic option for CD44v6-expressing malignancies. However, it could be related that the limited reduction of tumor burden in the group treated with CD44v6-CAR-NK92 cells may be due to the short lifetime and low persistence of irradiated NK92 cells in vivo. These results are indicative that future studies should include a method for improving NK92 cell persistence, such as cytokine support or other engineering approaches, to further improve their therapeutic efficacy.

The advantages of NK92 cells, particularly their standardization in production and simplification of regulatory requirements, are especially relevant in the context of the previously mentioned challenges. Thanks to their clonality, easy expansion, and the absence of patient-specific variables, NK92 cells offer significant benefits [62]. Moreover, NK cells do not cause GvHD, which is one major challenge observed in allogenic Tcell therapies [63]. In contrast to NK92 cells, autologous and allogeneic NK cells persist longer in vivo and can cause a more vigorous tumor-directed immune response by interacting with the natural immune system [64]. This may improve their potency against solid tumors partly because of their ability to persist within the tumor microenvironment and to attract endogenous immune cells to the tumor site [65]. The best cell source is thus related to the specific clinical application, but CAR-NK92 cells are an attractive choice due to ease of production and suitability for off-the-shelf therapies.

### Study limitations and future considerations

While our study demonstrated the in vivo efficacy of CD44v6-CAR-NK92 cells, several limitations should be addressed to enhance the clinical feasibility of this approach. One key limitation is the need to expand in vivo data across different cancer types. Although our results confirm the effectiveness of CD44v6-CAR-NK92 cells, further in vivo validation in other solid tumor models, such as breast cancer, pancreatic cancer, and glioblastoma, is crucial, given the strong in vitro responses observed. Additionally, testing patient-derived tumor samples with naturally expressed CD44v6 would provide a more clinically relevant assessment of therapeutic potential. Another critical factor is the optimization of NK92 cell administration schedules. Since irradiated NK92 cells have a limited lifespan in vivo, their therapeutic effect is short-lived. To enhance efficacy, future studies should explore higher dosing frequencies or multiple administrations per week, ensuring prolonged cytotoxic activity against tumors. A further challenge is the lack of a clinically relevant benchmark therapy for solid tumor targeting cell therapies. This makes comparative analysis difficult and limits the ability to contextualize the observed therapeutic effects. To address this, future research could incorporate alternative control groups, such as primary NK cells, T cells, or iPSC-derived effector cells, to better evaluate the efficacy of CD44v6-CAR therapy in a competitive setting. Despite these limitations, our study provides a strong foundation for the further development of CD44v6-CAR-based therapy in solid tumors. By addressing these challenges through optimized study designs and broader tumor model evaluations, future research can pave the way for the clinical translation of this promising therapeutic approach.

## Conclusion

Our study demonstrates that CD44v6-CAR-NK92 cells are effective against solid tumor models of various cancers both in vitro and in vivo. These findings illustrate the broad potential of CD44v6-CAR NK cells as a therapeutic option for solid tumors and suggests future pre-clinical and clinical studies to evaluate safety and effective dosage regimes.

## Electronic supplementary material

Below is the link to the electronic supplementary material.

Supplementary Material 1

Supplementary Material 2

Supplementary Material 3

## Data Availability

No datasets were generated or analysed during the current study.

## Abbreviations

AML: Acute myeloid leukemia
BLI: Bioluminescence imaging
CAR: Chimeric antigen receptor
DMEM: Modified eagle’s medium
ELISA: Enzyme-linked immunosorbent assay
hiFBS: heat inactivated Fetal Bovine Serum
FDA: The food and drug administration
GvHD: Graft-versus-Host Disease
IARC: The international agency for research on cancer
IFN-γ: Interferon-gamma
i.p.: Intraperitoneally
MCTS: Multicellular tumor spheroids
MM: Multiple myeloma
NEAA: Non-essential amino acid
NK: Natural killer
OD: Optical density
PBS: Phosphate buffered saline
PEI: Polyethylenimine
PS: Penicillin-streptomycin
RPMI: Roswell park memorial institute
TNBC: Triple negative breast cancer
UKCCCR: United kingdom coordinating committee on cancer research
