# Supplementary material for: Chimeric Antigen Receptor (CAR)-NK92 cells effective against glioblastoma, breast- and pancreatic cancer in vitro and in a murine xenograft model of ovarian cancer
Source: Cancer Cell Int. 2025 Jul 11;25:260. doi: 10.1186/s12935-025-03865-0 (PMC12255008; doi:10.1186/s12935-025-03865-0)
Supplement: Supplementary file 2 — Supplementary Material 2 [file 12935_2025_3865_MOESM2_ESM.docx]

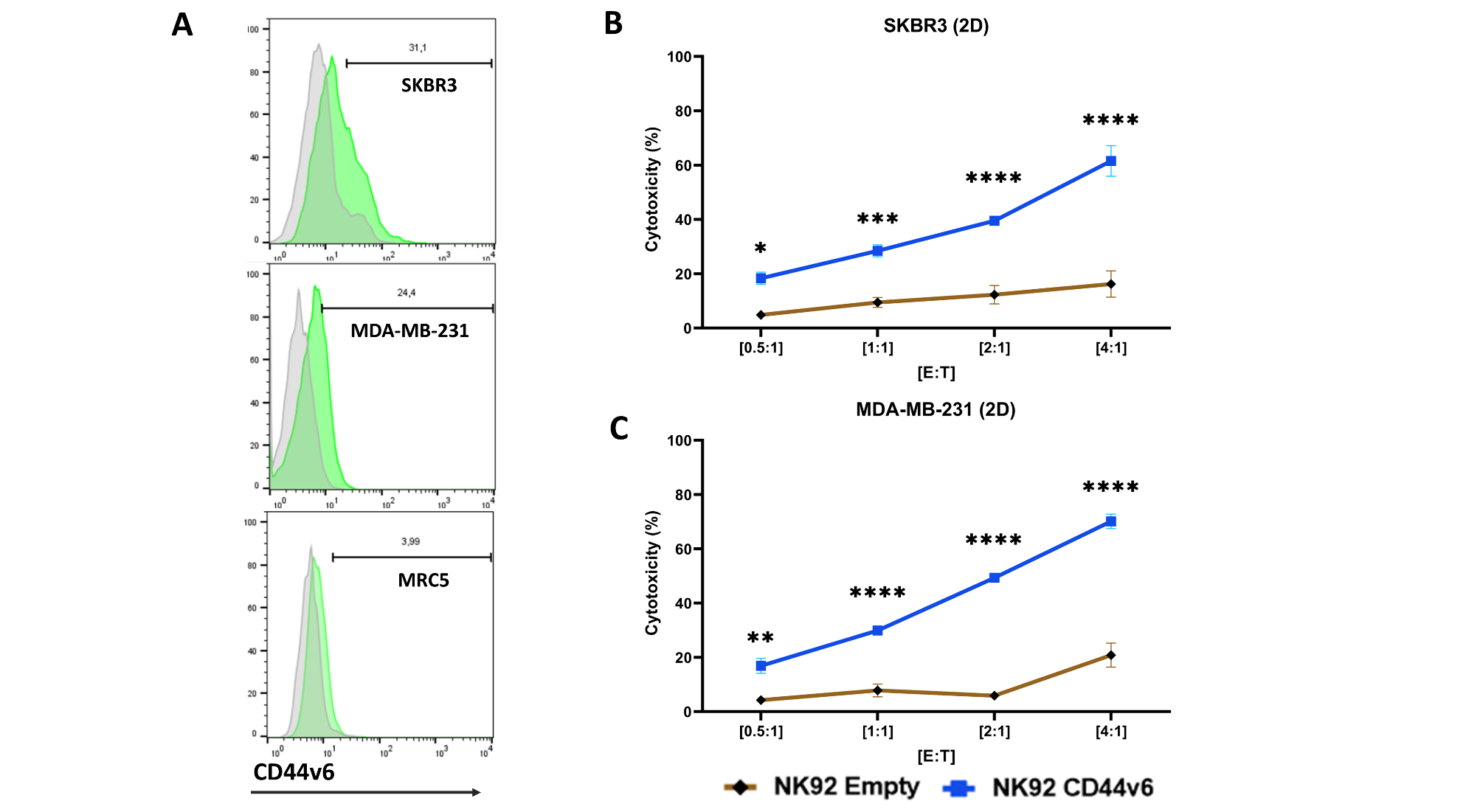


**Supplementary Figure 1. 2D model of cytotoxicity efficacy of CD44v6-CAR-NK92 against breast cancer:**

**A)** Representative flow cytometric analysis of CD44v6 expression on the breast cancer cell lines SKBR3, MDA-MB-231 and the fibroblast MRC5. **B-C)** Cytotoxicity assays showing the percent cytotoxicity of CD44v6-CAR-NK92 cells against breast cancer cell lines in the 2D model compared to Empty-CAR-NK92 cells at various effector-to-target ratios (E:T ratios) after 18 hours of incubation. The results represent n= 3-4 independent experiments performed in triplicate. Statistical significances are presented using two-way ANOVA and marked p < 0.05 = *, p < 0.01 = **, p < 0.001 = ***, p < 0.00001 = ****, and ns for non-significant p-values.


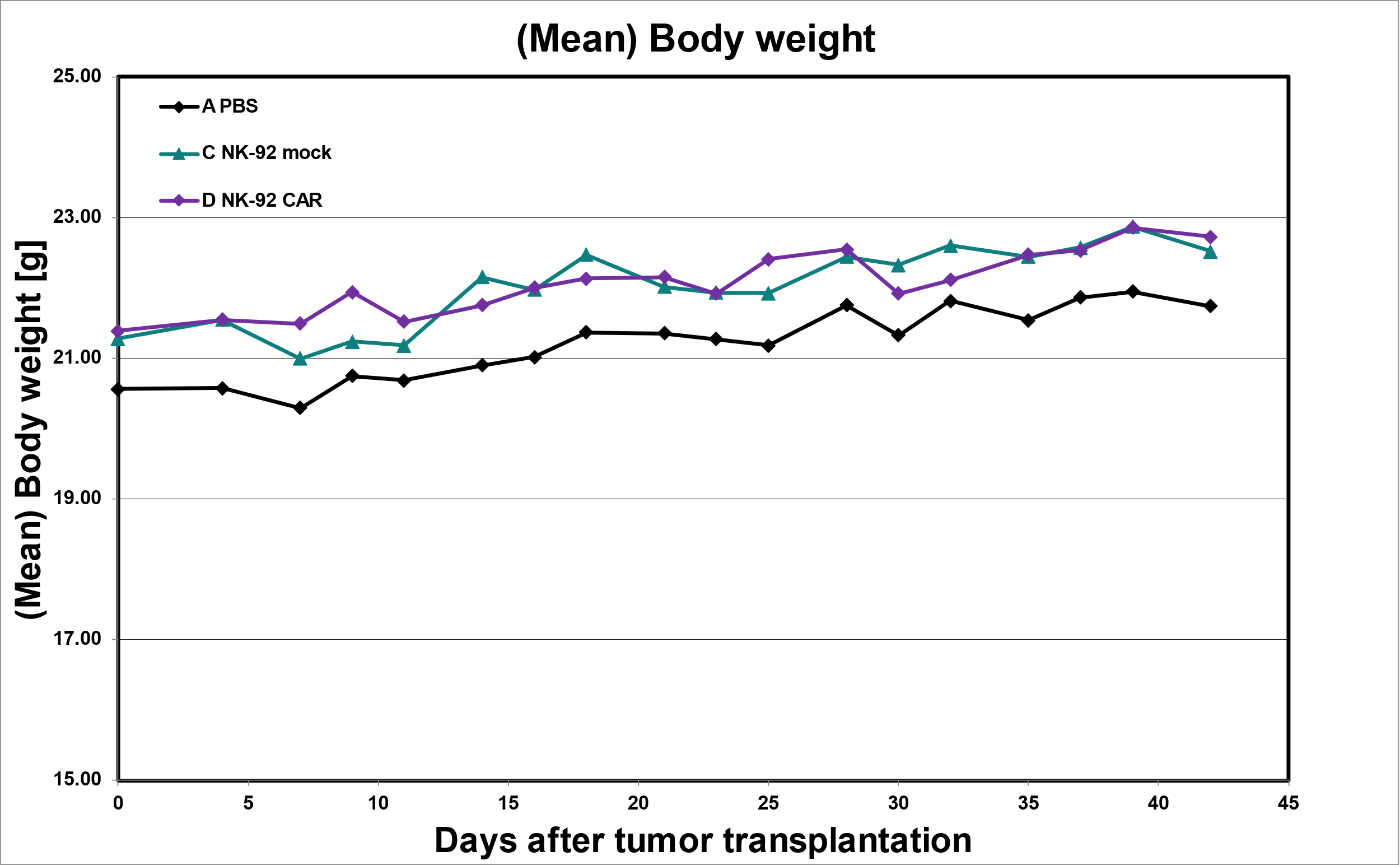


**Supplementary Figure 2. Mean body weight diagram of in vivo xenograft murine model:**

Graph illustrating the mean body weight changes (in grams) over time (day 0 to day 42) for all treatment groups. Treatment groups include PBS, NK92 Mock (Empty CAR NK92 cells transduced with a non-binding CAR), and NK-92 CAR (NK92 cells transduced with a CD44v6 CAR), providing an overview of treatment tolerability and general health across the study duration.
