# Supplementary material for: Chimeric Antigen Receptor (CAR)-NK92 cells effective against glioblastoma, breast- and pancreatic cancer in vitro and in a murine xenograft model of ovarian cancer
Source: Cancer Cell Int. 2025 Jul 11;25:260. doi: 10.1186/s12935-025-03865-0 (PMC12255008; doi:10.1186/s12935-025-03865-0)
Supplement: Supplementary file 3 — Supplementary Material 3 [file 12935_2025_3865_MOESM3_ESM.docx]

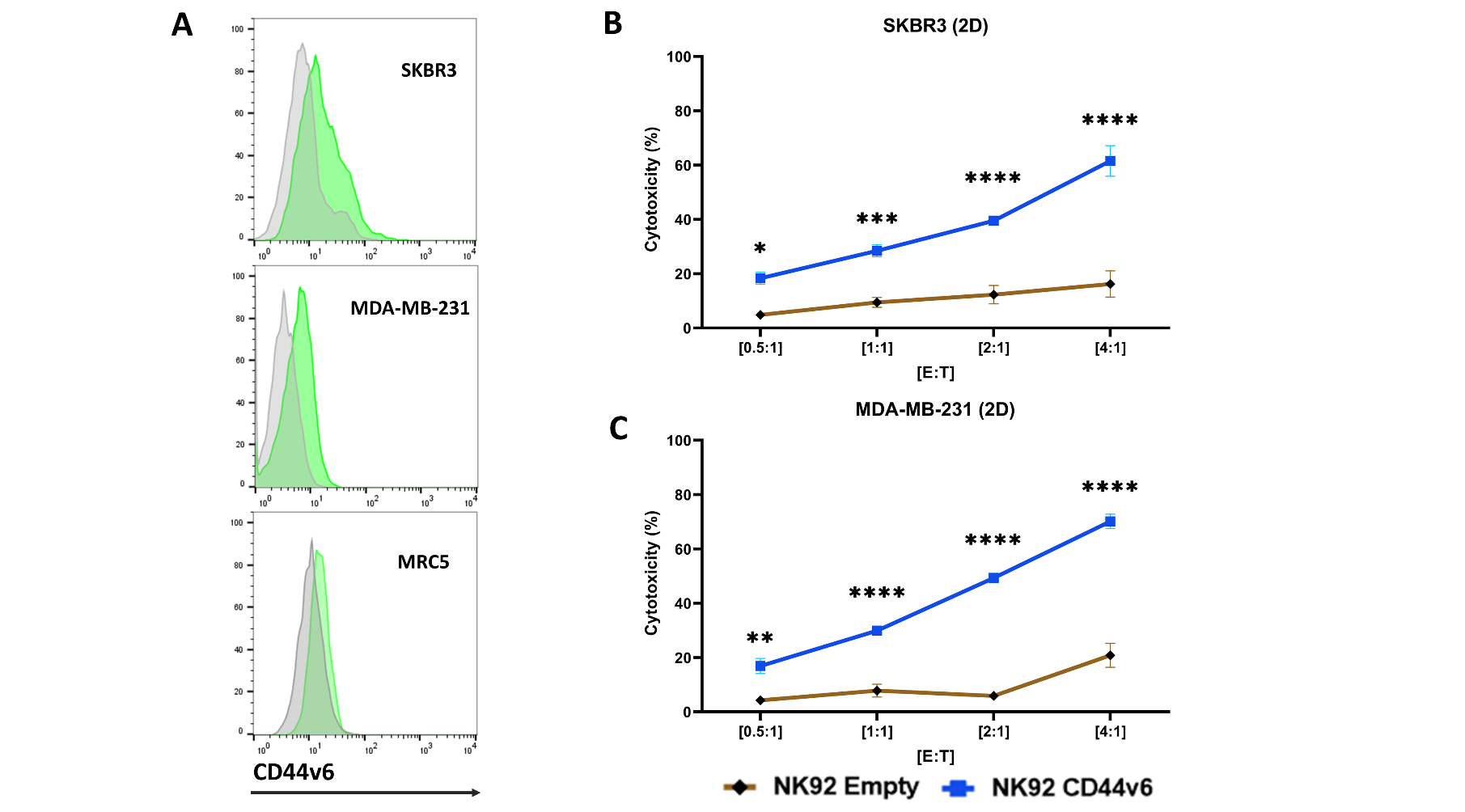


**Supplementary Figure 1. 2D model of cytotoxicity efficacy of CD44v6-CAR-NK92 against breast cancer:**

**A)** Representative flow cytometric analysis of CD44v6 expression on the breast cancer cell lines SKBR3, MDA-MB-231 and the fibroblast MRC5. **B-C)** Cytotoxicity assays showing the percent cytotoxicity of CD44v6-CAR-NK92 cells against breast cancer cell lines in the 2D model compared to Empty-CAR-NK92 cells at various effector-to-target ratios (E:T ratios) after 18 hours of incubation. The results represent n= 3-4 independent experiments performed in triplicate. Statistical significances are presented using two-way ANOVA and marked p < 0.05 = *, p < 0.01 = **, p < 0.001 = ***, p < 0.00001 = ****, and ns for non-significant p-values.


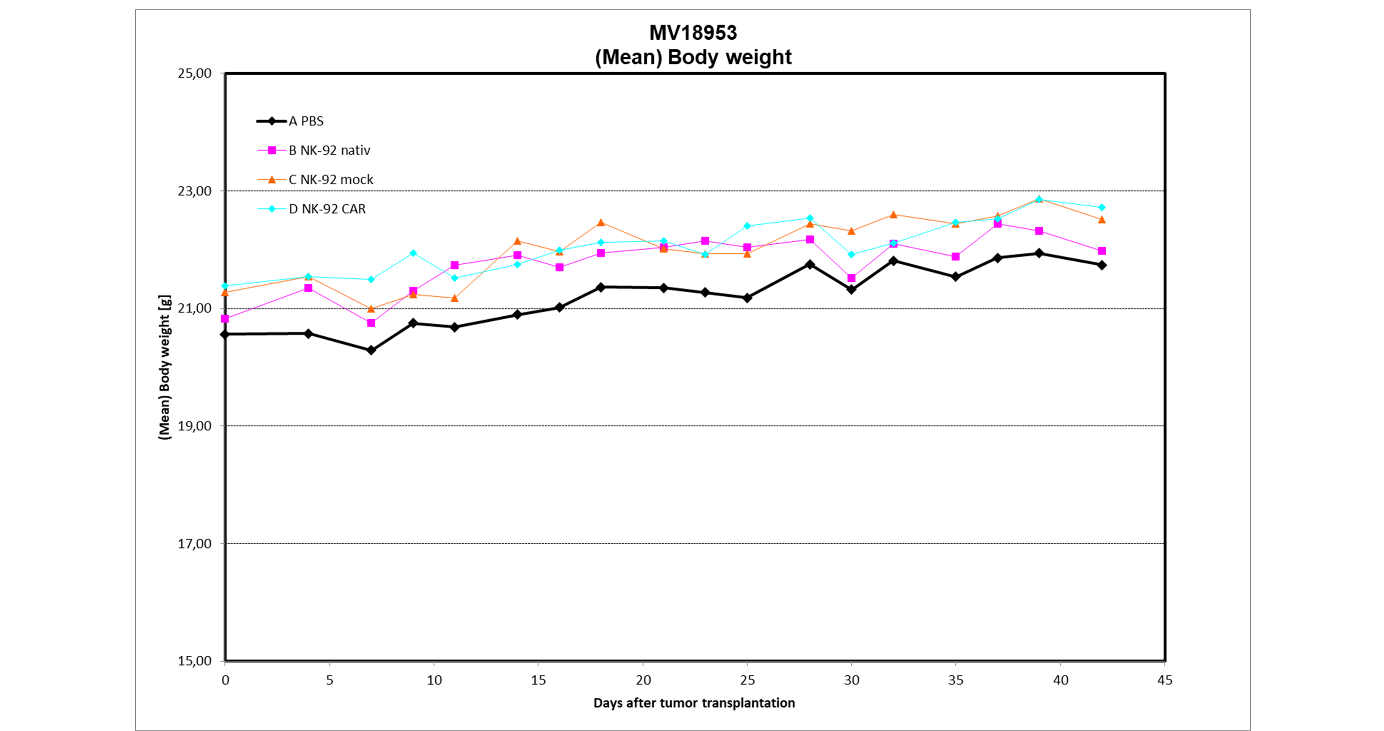


**Supplementary Figure 2. Mean body weight diagram of in vivo xenograft murine model:**

Graph illustrating the mean body weight changes (in grams) over time (day 0 to day 42) for all treatment groups. Treatment groups include PBS, NK92 native, NK92 Empty, and NK92-CD44v6 CAR, providing an overview of treatment tolerability and general health across the study duration.

**Supplementary Table 1. Individual body weight table of xenografted murine model:**

Table presenting the individual body weights (in grams) of each mouse across 18 measurement points, grouped by treatment. Treatment groups include Group A (PBS), Group B (NK92 native), Group C (NK92 Empty), and Group D (NK92-CD44v6 CAR).

**
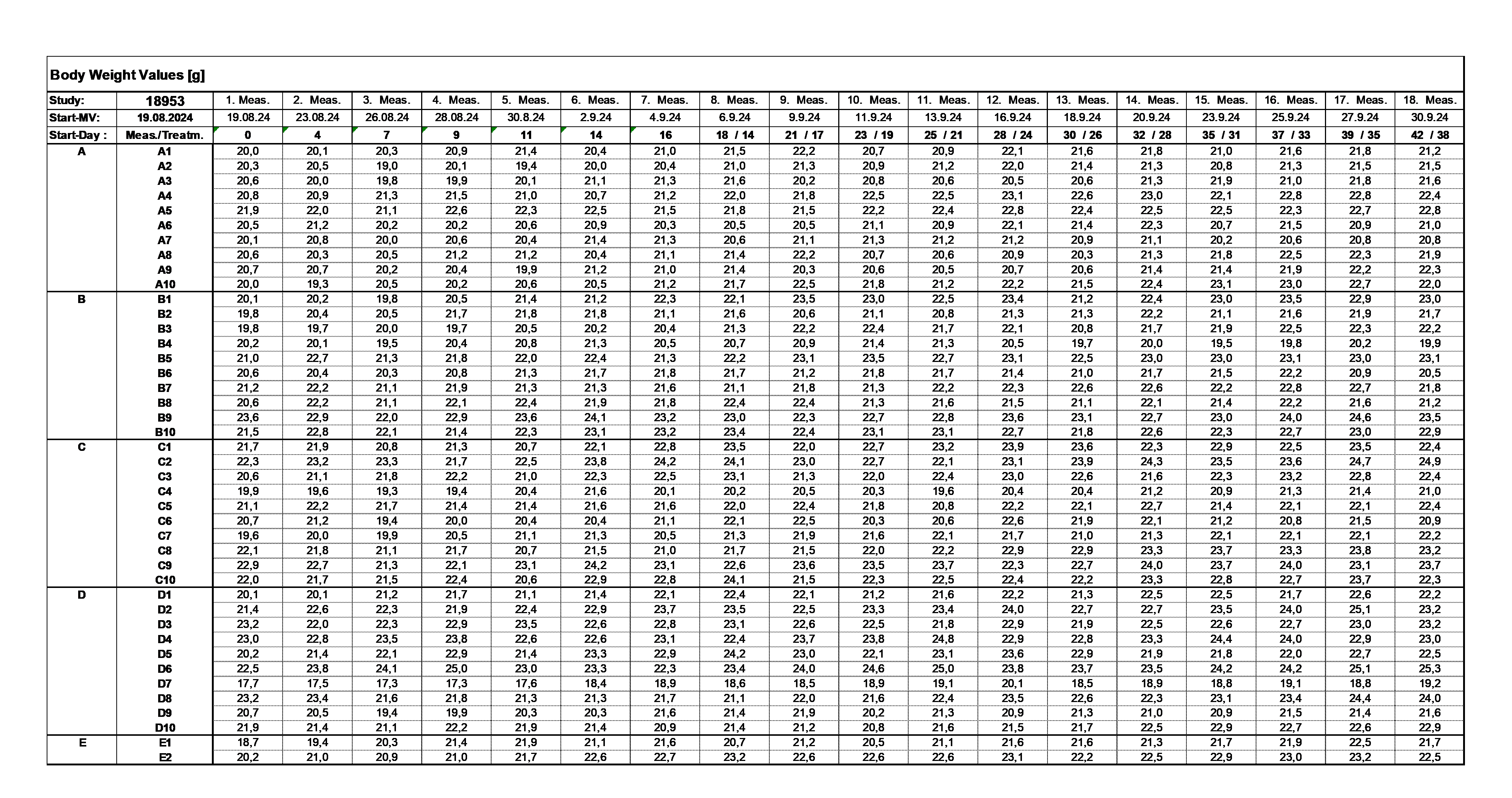
**
